# Quiz Questions

**Published:** 1885-10

**Authors:** 


*Quiz Questions.*—Course on Dental Pathological and Therapeutics, Philadelphia Dental College. Prof. J. Foster Flagg, D. D. S. Answered by William C. Foulks, D. D. S. Third edition, Revised and Enlarged. Publishers: The S. S. White Dental Manufacturing Company, Philadelphia, New York, Boston and Chicago.

This excellent series of questions and answers on Dental Pathology and Therapeutics is again offered to the dental profession as a work of reference in daily office practice, and is the only book that contains in a condensed and practical form the facts and principles of these subjects, as enunciated by Professor Flagg. Commencing with General Principles, these questions and answers relate to Deciduous Teeth, Pathological Dentition, Lancing, Permanent Teeth, Dental Caries, Sensitive Dentine, Galvanic Action, Pulp Protectors, Obtunding Applications, Pulp Capping, Pathological Conditions of Pulp, Dental Exostosis, Malformed Teeth, Periodontitis and Alveolar Abscess, the whole constituting a work of great value to all engaged in the practice of dentistry. The work is interleaved with blank pages for notes, etc., and gotten up in a neat and excellent style. We take great pleasure in commending this treatise as a valuable adjunct to the regular text-books of the profession.

